# Sensory neuronopathy complicating systemic lupus erythematosus: a case report

**DOI:** 10.1186/1752-1947-8-141

**Published:** 2014-05-07

**Authors:** Mitrakrishnan Rayno Navinan, Paramarajan Piranavan, Ali Uthuman Ali Akram, Jevon Yudhishdran, Thambyaiah Kandeepan, Aruna Kulatunga

**Affiliations:** 1 National Hospital of Sri Lanka, Regent Street, Colombo, Sri Lanka

**Keywords:** Dorsal root ganglionopathy, Intravenous immunoglobulins, Sensory neuronopathy, Systemic lupus erythematosus

## Abstract

**Introduction:**

Systemic lupus erythematosus is a multi-system connective tissue disorder. Peripheral neuropathy is a known and underestimated complication in systemic lupus erythematosus. Ganglionopathy manifests when neuronal cell bodies in the dorsal root ganglion are involved. Autoimmune disorders are a known etiology, with systemic lupus erythematosus being a rare cause.

**Case presentation:**

A 32-year-old South Asian woman presented with oral ulceration involving her lips following initiation of treatment for a febrile illness associated with dysuria. She had a history of progressively worsening numbness over a period of 4 months involving both the upper and lower limbs symmetrically while sparing the trunk. Her vibration sense was impaired, and her reflexes were diminished. For the past 4 years, she had had a bilateral, symmetrical, non-deforming arthritis involving the upper and lower limbs. Her anti-nuclear antibody and anti-double-stranded deoxyribonucleic acid status were positive. Although her anti-Ro antibodies were positive, she did not have clinical features suggestive of Sjögren syndrome. Nerve conduction studies revealed sensory neuronopathy. A diagnosis of systemic lupus erythematosus complicated by sensory neuronopathy was made. Treatment with intravenous immunoglobulin resulted in clinical and electrophysiological improvement.

**Conclusion:**

Peripheral neuropathy in systemic lupus erythematosus can, by itself, be a disabling feature. Nerve conduction studies should be considered when relevant. Neuropathy in systemic lupus erythematosus should be given greater recognition, and rarer forms of presentation should be entertained in the differential diagnosis when the clinical picture is atypical. Intravenous immunoglobulin may have role in treatment of sensory neuronopathy in systemic lupus erythematosus.

## Introduction

Systemic lupus erythematosus (SLE) is a connective tissue disorder that has the potential to cause disease in more than one system. It may involve the autonomic, peripheral or central nervous system. Though not given prominence by clinicians, the peripheral nervous system involvement could significantly affect patients’ quality of life [1]. Peripheral neuropathy is known to occur in SLE at rates anywhere between 5% and 27% [2], but a sizeable number of SLE patients (>50%) have subclinical nervous system involvement detected only by nerve conduction studies (NCSs) [3]. Conversely, affected small-diameter nerves will give rise to symptoms even in the absence of NCS findings. Peripheral nervous system involvement is considered a late feature [4] and is found with greater prevalence in patients with central nervous system involvement and in those with high Systemic Lupus Erythematosus Disease Activity Index scores. However, this perspective is at times controversial and not agreed upon [1,3]. The exact pathophysiology of peripheral neuropathy is unclear, and various suggestions have been offered as to its origin, including mediation through anti-neuronal antibodies, anti-cardiolipin antibodies and vasculitis with immune complex deposition and subsequent damage [5,6]. In patients with SLE, peripheral neuropathy often occurs as a mild-natured, distal, symmetrical sensorimotor or sensory neuropathy, and less so as severe and symptomatic mononeuritis multiplex and acquired demyelinating polyneuropathy similar to that of acute or chronic inflammatory demyelinating polyneuropathy [4,7,8]. Peripheral neuropathy in SLE patients is rarely seen as a plexopathy [5] or sensory neuronopathy [9]. Sensory neuronopathy causes a pure sensory disorder because of the involvement of sensory neurons within the dorsal root ganglion [10]. The degeneration is associated with an inflammatory T-cell reaction driven mainly by a cell-mediated immune response [11]. All sensory aspects maybe affected, but proprioception and vibration are predominantly impaired and the motor system is spared [12]. Other signs include unsteady gait, pseudo-athetoid movements of the hand [13], ataxia, areflexia and allodynia [12]. Although relatively rare, sensory neuronopathy should be included in the differential diagnosis of predominantly sensory or ataxic neuropathies [12]. In our present report, we describe the case of a patient with SLE who presented with peripheral neuropathy secondary to sensory neuronopathy and demonstrated an encouraging response to intravenous immunoglobulin (IVIG) treatment.

## Case presentation

A 32-year-old South Asian woman presented to our hospital with sudden-onset development of mouth ulcers involving her oral cavity and lips following treatment for a febrile episode associated with dysuria. She had a 4-year prior history of recurrent symmetrical painful swelling of small and large joints involving both upper and lower limbs without deforming arthropathy. She had no symptoms or signs during that period. In the resource- and investigation-limited peripheral hospital setting in Sri Lanka, she was diagnosed as having seronegative rheumatoid arthritis and treated accordingly. Episodic relapses were managed symptomatically with non-steroidal anti-inflammatory agents and disease-modifying anti-rheumatic drugs. During the 6 months preceding her presentation to our hospital, she had a significant weight loss of 7kg and developed numbness involving the lower and upper limbs symmetrically over a period of 4 months, which caused significant impairment of her activities of daily living. Her symptoms progressively worsened, especially following the current episode that led her to present to our hospital. Her presenting complaint was attributed to hypothyroidism by the previous investigating team, which found an elevated thyroid-stimulating hormone level during her period of illness (8.97μIU/ml (normal range, 0.497 μIU/ml to 4.5 μIU/ml)). Following initiation of treatment of a suspected urinary tract infection due to symptoms of fever and dysuria supported by urinalysis findings, she developed mouth ulcers, which were considered a drug reaction. While under investigation and treatment, the patient developed an acute painful swelling of the right calf which was confirmed to be deep vein thrombosis (DVT). She had no history of xerostomia or xerophthalmia at any given time during the disease course. She had no high-risk behavior or habits such as alcohol consumption or smoking.

A general examination revealed that she was febrile and had conjunctival edema and injection with oral mucosal ulceration. Additionally, she had erythematous macules and targetoid lesions over her face, palms and soles. The lesions were progressive and involved her chest in the form of erythematous dusky papules and patches. No lymph nodes were observed. Her cardiovascular, respiratory and abdominal examinations were normal. A peripheral nervous system examination revealed diminished vibration sense in both upper and lower limbs, and joint proprioception was preserved. Two-point discrimination distance was abnormal in both upper and lower limbs (palms: 3cm on the right side and 2cm on the left side (normal range, 0.8cm to 1.5cm); shins: 6cm bilaterally (normal range, 3cm to 4cm)). Her stereognosis was impaired, but her graphesthesia remained intact. Although her reflexes were diminished, her pain and soft touch remained unaffected. Her muscle power and tone were normal. The central nervous system findings were normal.

**Figure 1:**
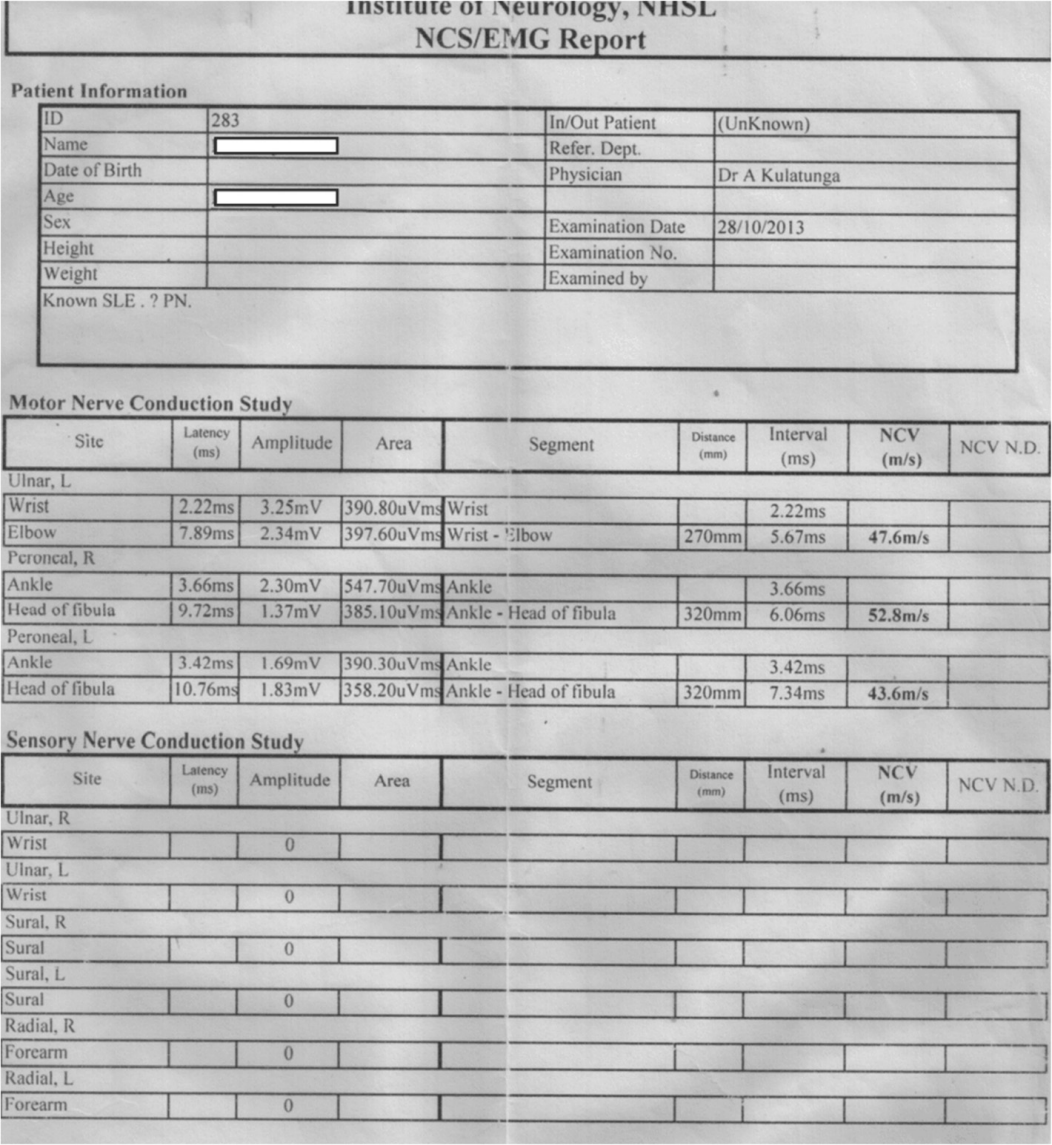
**Patient summary report of preliminary nerve conduction study taken at time of presentation shows electrophysiological parameters suggestive of sensory neuronopathy.** [NCV-nerve conduction velocity, EMG-electromyogram, NCS-nerve conduction study, NCV N.D- nerve conduction velocity normal distribution, SLE- systemic lupus erythematosus, PN- peripheral neuropathy, R- right, L - left].

An investigation showed that the patient had an elevated erythrocyte sedimentation rate (ESR) of 88mm for the first hour. However, her C-reactive protein level was normal at 0.6mg/L (normal range, <5mg/L). Hematology examination revealed a bicytopenia, with a white blood cell count of 1.74×10^9^/L, a hemoglobin level of 8.8g/dl (normal range, 11g/dl to 18g/dl) and a normal platelet count of 308×10^9^/L. Repeated counts showed similar findings. A bone marrow biopsy showed mild hypocellularity with active hematopoiesis. Her serum iron level was normal at 87μg/dl (normal range, 37μg/dl to 148μg/dl). She had a marginally low normal total iron binding capacity value of 274μg/dl (normal range, 274μg/dl to 386μg/dl) and normal iron saturation of 31.7% (normal range, 15% to 50%). Her serum ferritin was elevated at 1,100μg/L (normal range, 12μg/L to 190μg/L). Her alanine and aspartate aminotransaminase levels were elevated at 81IU/L (normal range, 10IU/L to 35IU/L) and 79 IU/L (normal range, 10IU/L to 40IU/L), respectively, but her other liver parameters were within reference ranges. Her coagulation screening results were normal, including activated partial thromboplastin time. Repeat thyroid function tests were within reference ranges. Her direct Coombs’ agglutination test was negative. Her anti-nuclear antibody and anti-double-stranded deoxyribonucleic acid (DNA) antibody were both positive. Her anti-cardiolipin antibodies were positive and moderately elevated at 57.9 (normal range, <15), but the repeat value 12 weeks later was normal. Her complement levels showed a marginally low normal complement component 3 (C3) value of 55.4mg/dl (normal range, 55mg/dl to 120mg/dl) and a reduced C4 level of 11mg/dl (normal range, 20mg/dl to 50mg/dl). Her renal function was normal with a serum creatinine of 0.71mg/dl (normal range, 0.50mg/dl to 1.10mg/dl). Urinalysis demonstrated the presence of protein, and quantification demonstrated a urinary protein level of 139.1mg/dl, a urinary creatinine level of 68.4mg/dl and a urine protein/creatinine ratio of 2.08 (normal range, <0.4). An ultrasound of her abdomen was normal and demonstrated normal-sized kidneys. A renal biopsy revealed class III lupus nephritis. Retroviral screening and venereal disease research laboratory screening were negative. Anti Ro antibodies were found to be positive. Hepatitis B surface antigen and hepatitis C virus (HCV) antibodies were both negative. Epstein-Barr virus (EBV) immunoglobulin M (IgM) antibodies were positive. NCS revealed a sensory neuronopathy (Figure 1). The two-dimensional echocardiogram, electrocardiogram and chest X-ray were normal.

**Figure 2:**
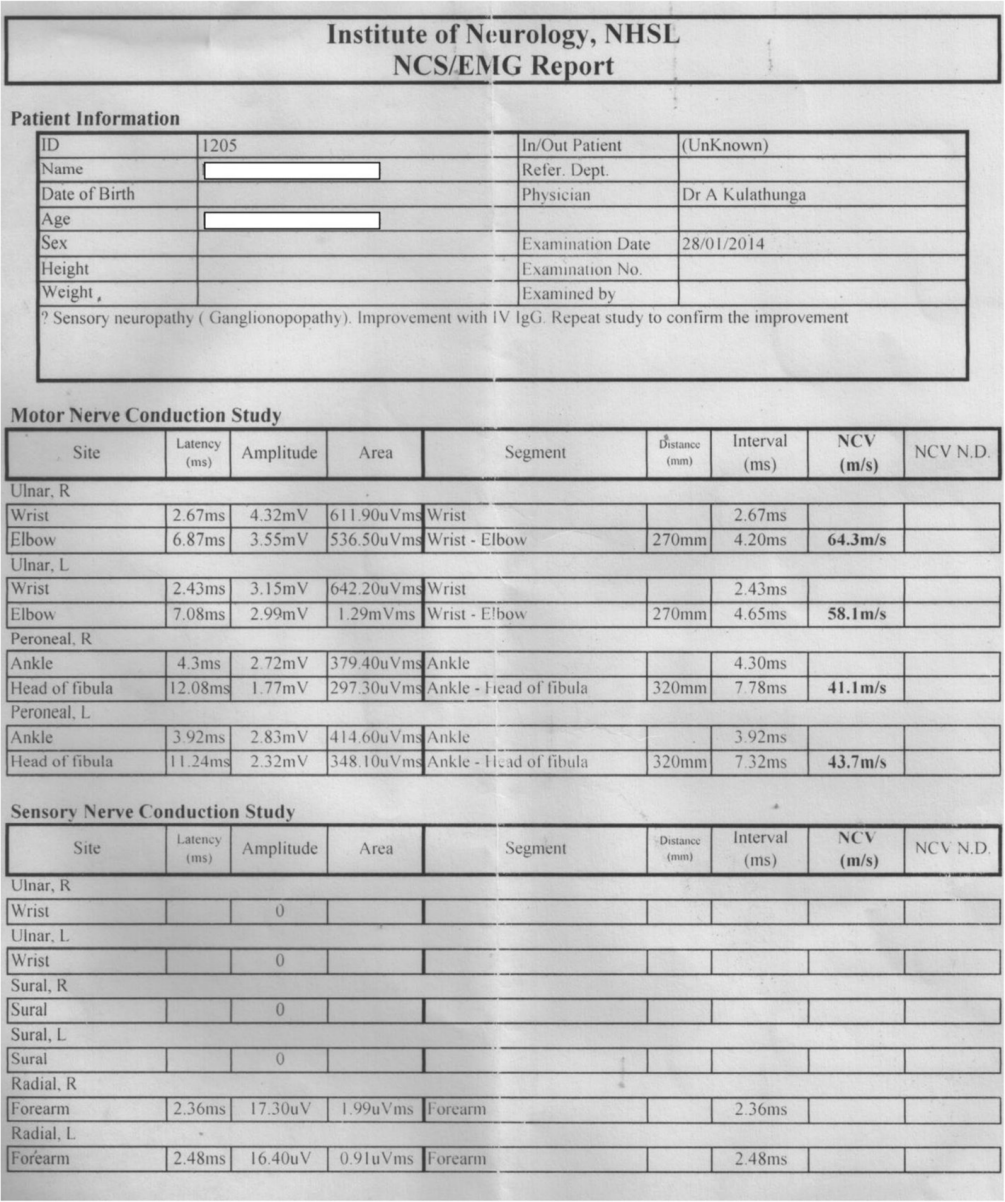
**Post intravenous immunoglobulin nerve conduction study shows relative improvement in sensory electrophysiological parameters in comparison to preliminary findings of sensory neuronopathy.** [NCV-nerve conduction velocity, EMG-electromyogram, NCS-nerve conduction study, NCV N.D- nerve conduction velocity normal distribution, IVIG- intravenous immunoglobulin, R- right, L - left].

The patient was started on immunosuppressive treatment with high-dose oral prednisolone (1mg/kg/day), which was slowly tapered down; mycophenolate mofetil (250mg twice daily); and warfarin for anti-coagulation for her DVT. She was examined 2 months later at a follow-up appointment, but, owing to persistence of sensory neuronopathy without improvement, a clinical decision was made to give a her trial of IVIG (0.4gm/kg/day) for 5 days. Upon clinical reassessment 4 weeks later, she showed improvement. NCSs were repeated, and the findings (Figure 2) partially corroborated the observed clinical improvement. Hematological parameters also showed clinical improvement, with gradual reduction of her ESR. Her bicytopenia had regressed, and repeat whole-blood analysis revealed only mild persistent anemia with a normal white blood cell count.

## Discussion

In our present report, we describe the case of a patient with a high Systemic Lupus Erythematosus Disease Activity Index score of 26 (scores >12 define a severe flare) [14] complicated by DVT. The patient’s bicytopenia was probably a complication of autoimmune bone marrow suppression, as glucocorticoid treatment caused quick reversal of the blood analysis. EBV could have been a subclinical infection responsible for the patient’s IgM positivity, but it could also have been an incidental finding. The disabling (Additional file 1: Video 1) presentation of numbness (Barthel Index activities of daily living score 45 of 100 [15]) prompted further investigation. The findings were that of a sensory neuronopathy, which is a known complication of Sjögren syndrome (SS) but an uncommon manifestation of SLE.

Sensory neuronopathy is known to occur secondary to neoplastic disorders, as a drug-induced phenomenon (for example, as a reaction to cisplatin or vitamin B6) [16,17], in inherited disorders with degeneration of dorsal root ganglion cells, following viral infections (for example, human immunodeficiency virus (HIV), HCV, EBV) [18-20], as an idiopathic phenomenon and in autoimmune diseases (for example, SS, Miller Fisher syndrome and Bickerstaff’s brainstem encephalitis, chronic active hepatitis, and gluten sensitivity) [13,21,22]. There is no reference standard for diagnosing peripheral neuropathies. For sensory neuronopathy, various modalities have been suggested and tried. Magnetic resonance imaging (MRI) can be useful, but is non-specific and has technical issues that may cause variability results. Skin biopsy is also useful, as it may demonstrate non-length-dependent involvement, especially in difficult situations, but it is considered invasive and can be done only in specialized centers. In patients with sensory neuronopathy, a common clinical and electrophysiological pattern distinctively differentiates it from other sensory neuropathies. Therefore, NCS findings can be very useful in the differential diagnosis, though MRI and NCS findings in combination can be considered virtually diagnostic [11,12,23]. In patients with sensory neuronopathy, neurophysiological abnormalities are dominated by widespread decrease in sensory nerve action potential amplitudes involving both upper and lower limb nerves, even with an asymmetrical or patchy distribution. This non-length-dependent pattern of peripheral axonal degeneration with impairment of sensory nerve conduction should be considered the hallmark of neuronopathies [23].

EBV can also result and present with a vast spectrum of neurological manifestations, as it can involve the whole neural axis, from central to peripheral, and affect any part of the nervous system [24]. Positive serological findings may be incidental because IgM antibodies are known to remain positive for as long as 1 year. Though EBV cannot be conclusively ruled out as a cause of neuronopathy, in the absence of symptomatic infection, as reported by Rubin *et al*. [20], SLE rather than EBV is likely to have been the main cause underlying our patient’s presentation.

The pathophysiological mechanism of neuropathy varies, depending on the type, as does the treatment recommended to relieve patients’ symptoms [5]. Although treatment with glucocorticoids, plasma exchange and IVIG alone or in combination with mycophenolate mofetil have been tried and suggested, the supporting evidence is anecdotal at best because none of these regimens have been studied in a controlled manner. There is also the possibility of spontaneous stabilization and resolution [14,25]. Chimeric monoclonal antibodies such as rituximab and infliximab have also been found to be useful [26,27]. IVIG has been used successfully to treat many neurological conditions effectively, either alone or in combination with other agents [28]. It has also been shown to be beneficial in the treatment of sensory neuronopathy, even in patients with chronic conditions; however, SS was the underlying disease in previously reported cases [29]. The use of IVIG to treat sensory neuronopathy due to SLE is poorly described in the medical literature. As the available evidence is based only on case reports and clear protocols are lacking, questions arise as to the strength of the evidence to support current treatment options. In our patient, the level of her disability warranted intensive treatment because her sensory neuronopathy did not show spontaneous improvement or an acceptable response to high-dose prednisolone and mycophenolate mofetil. A trial of IVIG at 0.4gm/kg/day was given for a period 5 days. When the patient was followed up 1 month later, she demonstrated clinically definitive improvement (Barthel Index activities of daily living score 70 of 100) and relative improvement in two-point discrimination of the upper limbs, 2cm on the right palm and 1.8cm on the left palm (normal range, 0.8cm to 1.5cm). The electrophysiological parameters of NCS supported these improvements. Our patient’s response to treatment is in keeping with previous successful attempts to treat neuronopathy [14,29]. Two further cycles of IVIG are pending in our patient, though cost is a limiting factor. Even though evidence is based predominantly on case reports, the positive response to IVIG merits recognition and further study, because, to the best of our knowledge, this report describes the first case with encouraging results in which IVIG was trialed in a patient with sensory neuronopathy due to SLE.

When SLE patients complain of neuropathy, attempts should be made to classify and identify the type of peripheral neuropathy. Care should be taken not to overlook the possibility of connective tissue disease as the etiology of peripheral neuropathy, because it may easily be attributed to other causes. Our patient’s clinical picture did not fully match a classical sensory neuronopathy, even though her motor system remained unaffected. This presentation demonstrates that, although the clinical features may point toward a sensory neuronopathy, investigations such as NCS are invaluable in helping to confirm a diagnosis when the presentation is atypical.

## Conclusions

Peripheral neuropathy in SLE, although poorly appreciated, can be a disabling manifestation. SLE patients should be screened routinely for neuropathy, even if they are asymptomatic, and, when neuropathy is present, NCS may help in identifying its type. Though uncommon, when pure sensory symptoms and clinical signs dominate, sensory neuronopathy should be considered in the differential diagnosis. IVIGs may have a role in the treatment of sensory neuronopathy, even in SLE patients.

## Consent

Written informed consent was obtained from the patient for publication of this case report and accompanying images. A copy of the written consent is available for review by the Editor-in-Chief of this journal.

## Abbreviations

DVT: Deep vein thrombosis; EBV: Epstein-Barr virus; IVIG: Intravenous immunoglobulin; NCS: Nerve conduction study; SLE: Systemic lupus erythematosus; SS: Sjögren syndrome.

## Competing interests

The authors declare that they have no competing interests.

## Authors’ contributions

All authors participated in making the clinical diagnosis. All authors provided care for the patient. MRN and AK researched and drafted the manuscript. All authors read and approved the final manuscript.

## Authors’ information

MRN is a registrar of medicine at the National Hospital of Sri Lanka, Colombo. PP and AUAA are intern medical officers at the National Hospital of Sri Lanka, Colombo. JY and TK are senior registrars in medicine at the National Hospital of Sri Lanka, Colombo. AK is a consultant physician in acute medicine at the National Hospital of Sri Lanka, Colombo.

## Supplementary Material

Additional file 1: Video 1 Taken prior to intravenous immunoglobulin therapy, the video demonstrates the level of disability, where the simple task of grasping and writing with a pen poses significant challenge due to sensory neuronopathy. Click here for file
